# Association between Healthy Eating Index-2015 and prostate enlargement: A cross-sectional study of the National and Nutrition Examination Survey 2001–2008

**DOI:** 10.29219/fnr.v68.10828

**Published:** 2024-08-27

**Authors:** Xing-peng Di, Chi Yuan, Xin Wei

**Affiliations:** 1 Department of Urology and Institute of Urology (Laboratory of Reconstructive Urology), West China Hospital, Sichuan University, Chengdu, Sichuan, People’s Republic of China; 2 Department of Pediatric Surgery, West China Hospital, Sichuan University, Chengdu, Sichuan, People’s Republic of China

**Keywords:** health eating index, prostate enlargement, National Health and Nutrition Examination Survey, vegetable, dairy

## Abstract

**Background:**

Benign prostate hyperplasia (BPH) occurs in elder men globally with high prevalence. Human diet and lifestyle aroused great attention in the prevalence of BPH. Prostate enlargement (PE) is a major symptom of BPH.

**Objectives:**

To elaborate the effect of total diet quality for adults from the United States, we investigated the association between Health Eating Index (HEI)-2015 and the risk of PE in adults from the National Health and Nutrition Examination Survey (NHANES).

**Methods:**

This cross-sectional study was conducted based on NHANES 2001–2008. Participants who reported a PE history were included. We conducted a logistic regression analysis to investigate the association between HEI-2015 and PE.

**Results:**

A total of 4,866 male participants aged 40 and above were enrolled. Compared with Q1 of HEI-2015, no significant differences were found in adjusted models. Higher vegetables intake (Odds ratio [OR] = 1.073; 95% confidence interval [95%CI] 1.015 to 1.134, *P* = 0.02) and higher total dairy intake (OR = 1.034; 95%CI 1.009 to 1.061, *P* = 0.01) were significantly related with higher risk of PE.

**Conclusions:**

There was no significant difference between HEI-2015 and PE after full adjustment. Total vegetables and dairy product might be associated with higher risk of PE and needed further validation.

**Popular scientific summary**

- Benign prospate hyperplasia (BPH) is a highly prevalent disease in elder men. Daily lifestyle and diet are regarded potential factors related to BPH.
- Although there is no significant association between HEI-2015 score and prostate enlargement (PE),total vegetables and dairy intake may be associated with the risk of PE.
- Further investigation of the association, especially causal relationship, between more nutritional and diet intake with BPH is warranted.

Benign prostate hyperplasia (BPH) occurs in elder men with a high incidence of more than 1/2 in those over 50 years old (1). The enlargement of prostate can be initiated before 30 years of age and ultimately causes lower urinary tract symptoms (LUTS). LUTS mainly includes urine storage period (i.e. hesitancy, awaiting, emergency, high frequency, and straining), voiding stage (i.e. interruption and weaken), and post-voiding stage (i.e. incessant urine) (2, 3). Hence, BPH leads to heavy health and economic burden to patients globally (4, 5). However, the mechanism of the occurrence of BPH has not been fully determined.

Human diet and lifestyle have changed significantly over the past several decades. Diet-associated BPH has therefore aroused great attention. Studies have demonstrated that diet was a vital factor influencing the risk of BPH (6). The prostate Cancer Prevention Trial has identified relationships between BPH and some nutrients, including lycopene, zinc, and vitamin D (7). The trial also revealed that fat and red meat consumption was associated with the progression of BPH. Furthermore, a case-control study from Italy revealed that cereals, bread, eggs, and poultry are risk factors contributing to BPH. On the contrary, soups, pulses, and cooked vegetables are identified as protective factors (8). However, red meat intake was also recognized as protective factor of BPH (9). Intriguingly, whether the consumption of vegetables and fruits had a major role in causing BPH is still under discussion and is therefore not strongly recommended by American Urological Association (AUA) and European Association of Urology (EAU) guidelines (10, 11). For instance, a study from the United States identified that vegetable intake is negatively associated with BPH, whereas fruit intake is not (11).

In order to elaborate the effect of total diet quality for adults from the United States, we introduced the Health Eating Index (HEI) for a better insight into the association between diet and prostate enlargement (PE). The HEI is based on Diary Guidelines for Americans (DGA) to assess the influence of food series quality on US elderly adults. HEI-2015 is an updated version corresponding to the 2015–2020 DGA with better validity and consistency (12). To date, no study has been conducted to analyze the association between PE and HE-2015 diet series. Hence, we performed the current study to illustrate whether the daily diet intake indicated by HEI-2015 affected PE development or not by using the data from the National Health and Nutrition Examination Survey (NHANES) 2001–2008. We aimed to provide evidence on the impact of a regular diet or its components on PE in males.

## Methods

### Study population

**Fig. 1:**
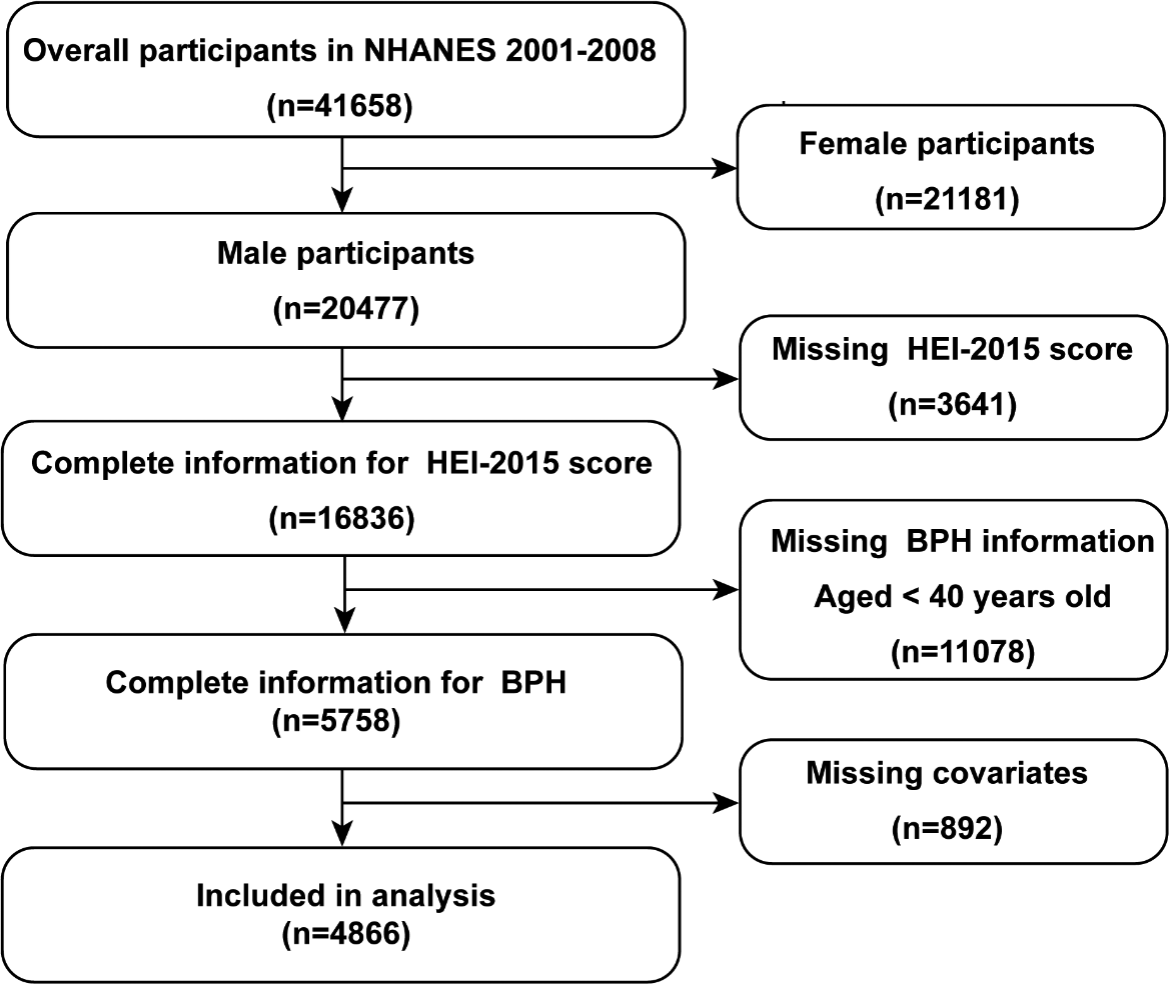
Flowchart of the study sample selection.

NHANES mainly included interviews and related examinations with 2 years for each cycle. In the current study, we introduced four cycles from 2001 to 2008 and included 41,658 participants aged 20 years or older. A total of 21,191 female participants were excluded. Then, 3,641 participants with a missing HEI-2015 score and 11,078 participants with missing PE data were excluded. After removing 892 data for missing covariates, 4,866 participants were included finally (Fig. 1). Notably, all the protocols were approved by the National Center of Health Statistics research ethics review board and all participants.

### Diet quality

The food intake data were retrieved from NHANES’s first 24 h recall interviews, which were performed by experienced interviewers. The first interview was conducted face-to-face in the Mobile Examination Center with more representatives and less missing data. The diet quality was measured by HEI-2015, which was scored from 0 to 100 and included 13 components (9 adequacy components and 4 moderation components). The components of the diet were evaluated based on the diet intake in the first 24 h and United States Department of Agriculture (USDA) MyPyramid Equivalents Database/Food Patterns Equivalents Database (MPED/FPED). These scores were incorporated to build a total score of 100.

### Assessment of PE

Prostate enlargement was the main outcome, which was ensured by the questionnaire ‘Have you ever been told by a doctor or health professional that you had an enlarged prostate gland?’ (KIQ121). The answers ‘Yes’ or ‘No’ were identified as having a history of PE or not through the self-reports of the participants.

### Covariates

To strengthen the association between HEI-2015 and PE, several confounding factors were collected from demographics (e.g. age, education level, and ethnicity), laboratory exams (e.g. diabetes), physical exams (e.g. body mass index [BMI]), and questionnaires (e.g. alcohol drinking). In detail, age was categorized as 40–50, 50–60, 60–70, 70–80, and over 80 years old. The race of the US population included Mexican American, non-Hispanic Black, non-Hispanic White, other Hispanic, and other races. Education level was labelled as ‘Less than 12th grade’, ‘High school grade’, and ‘College graduate’. Marital state included married, divorced, widowed, separated, never married, and living with partner states. The family income-to-poverty ratio was divided by 5. The body mass index (BMI, weight/standing height, kg/m^2^) was divided by 20, 25, and 30 kg/m^2^. The ‘*Yes/No*’ covariates belong to smoking, hypertension (average blood pressure over 140/90 mmHg), and coronary heart disease. The alcohol drinking included none (< 1 drink per week), light drinker (1–3 drinks per week), and heavy drinker (> 4 or more drinks per week). Diabetes was diagnosed based on the blood glucose tests conducted by doctors (13, 14). The categories of diabetes included diabetes mellitus, impaired fasting glucose (IFG), impaired glucose tolerance (IGT), and none.

### Statistical analysis

The sampling weights, strata and primary sample units were recommended by Centers for Disease Control and Prevention (CDC). The adjusted weight for the data should be a quarter of each cycle. The baseline data were presented as mean ± standard deviation (SD) for continuous variables and as proportions or counting numbers for categorial variables. The differences between groups were compared using the F-test for continuous variables and the Chi-square test for categorical variables. The Q1 was used as a reference.

Furthermore, multivariate logistic regression models with or without adjustment of covariates were applied to assess the odds of HEI-2015 on PE. In detail, model 1 was a crude model that was unadjusted. Model 2 was adjusted by age and race. Model 3 was adjusted by age, race, educational level, marital state, family income income-to-poverty ratio, BMI, smoke, energy, alcohol drinking, hypertension, diabetes, and coronary heart disease. In addition, the 13 components of HEI-2015 were analyzed by multinomial logistic regression model. In this process, the components were identified as continuous variables. Finally, stratified logistic analysis was conducted to identify the variables that modify the correlation between HEI-2015 and PE.

The sampling weights of 4 year cycles were applied under the guidance of the NHANES database (https://www.cdc.gov/nchs/nhanes/index.htm). The *R* software version 4.1 (http://www.R-project.org; The R Foundation) was used for data retrieving. The EmpowerStats (http://www.empowerstats.com, X&Y Solutions, Inc.) was utilized for further statistical analyses. A two-tailed *P* < 0.05 was considered as statistically significant.

## Results

### Baseline data description

There were 4,866 male participants aged 40 years and above who were included in this study (Fig. 1). The distribution and characteristics of the baseline data according to HEI-2015 quartiles were shown in Table 1. There were significant differences in age, energy, education level, marital state, family income-to-poverty ratio, hypertension, diabetes, and coronary heart disease compared to Q1 of HEI-2015. The higher quartile of the participants tended to be older, with higher educational level, and married. The higher HEI-2015 score is associated with higher prevalence rate of PE. However, there was no significant difference between BMI and HEI-2015 (Q2–Q4).

**Table 1:** Characteristics of participants by categories of Healthy Eating Index 2015 (HEI-2015): NHANES 2001–2008, weighted

Characteristics	Quartiles (Q) of HEI-2015				*P*
	Q1	Q2	Q3	Q4	
**Number**	1,130	1,275	1,217	1,244	
**HEI-2015**	36.0 (9.0–41.0)	46.0 (42.0–50.0)	55.0 (51.0–59.0)	66.0 (60.0–96.0)	<0.001
**Energy (kcal)**	2297.97 ± 1043.11	2364.17 ± 1087.73	2303.26 ± 1066.19	2201.36 ± 890.78	<0.001
**Age**	57.61 ± 12.60	59.04 ± 12.62	60.82 ± 13.37	62.73 ± 12.81	<0.001
**Race**					0.36
Mexican American	165 (14.60%)	214 (16.78%)	224 (18.41%)	233 (18.73%)	
Non-Hispanic Black	253 (22.39%)	269 (21.10%)	189 (15.53%)	214 (17.20%)	
Non-Hispanic White	645 (57.08%)	708 (55.53%)	710 (58.34%)	707 (56.83%)	
Other Hispanic	49 (4.34%)	54 (4.24%)	56 (4.60%)	64 (5.14%)	
Other Race	18 (1.59%)	30 (2.35%)	38 (3.12%)	26 (2.09%)	
**Education level**					<0.001
Less Than 12th Grade	394 (34.87%)	404 (31.69%)	354 (29.09%)	332 (26.69%)	
High School Grade	299 (26.46%)	322 (25.25%)	266 (21.86%)	244 (19.61%)	
College Graduate	437 (38.67%)	549 (43.06%)	597 (49.06%)	668 (53.70%)	
**Marital state**					0.002
Married	730 (64.60%)	890 (69.80%)	817 (67.13%)	909 (73.07%)	
Divorced	147 (13.01%)	140 (10.98%)	120 (9.86%)	114 (9.16%)	
Widowed	67 (5.93%)	74 (5.80%)	119 (9.78%)	75 (6.03%)	
Separated	36 (3.19%)	35 (2.75%)	31 (2.55%)	27 (2.17%)	
Never married	86 (7.61%)	79 (6.20%)	74 (6.08%)	72 (5.79%)	
Living with partner	64 (5.66%)	57 (4.47%)	56 (4.60%)	47 (3.78%)	
**Family income-to-poverty ratio**					0.006
≤ 4.99	888 (78.58%)	950 (74.51%)	869 (71.41%)	839 (67.44%)	
> 4.99	242 (21.42%)	325 (25.49%)	348 (28.59%)	405 (32.56%)	
**BMI (kg/m^2^)**					0.87
BMI ≤ 20	44 (3.89%)	45 (3.53%)	40 (3.29%)	34 (2.73%)	
20 < age ≤ 25	260 (23.01%)	330 (25.88%)	298 (24.49%)	304 (24.44%)	
25 < age ≤ 30	492 (43.54%)	515 (40.39%)	511 (41.99%)	530 (42.60%)	
30 < age	334 (29.56%)	385 (30.20%)	368 (30.24%)	376 (30.23%)	
**Smoke**					0.32
Non-smoker	786 (69.56%)	866 (67.92%)	795 (65.32%)	776 (62.38%)	
Smoker	344 (30.44%)	409 (32.08%)	422 (34.68%)	468 (37.62%)	
**Alcohol drinking**					0.003
Non-drinker	598 (52.92%)	584 (45.80%)	580 (47.66%)	541 (43.49%)	
Light drinker	265 (23.45%)	340 (26.67%)	258 (21.20%)	297 (23.87%)	
Heavy drinker	267 (23.63%)	351 (27.53%)	379 (31.14%)	406 (32.64%)	
**Hypertension**					0.18
No	586 (51.86%)	701 (54.98%)	680 (55.88%)	731 (58.76%)	
Yes	544 (48.14%)	574 (45.02%)	537 (44.12%)	513 (41.24%)	
**Diabetes**					0.09
DM	251 (22.21%)	260 (20.39%)	282 (23.17%)	289 (23.23%)	
IFG	74 (6.55%)	81 (6.35%)	80 (6.57%)	80 (6.43%)	
IGT	43 (3.81%)	19 (1.49%)	34 (2.79%)	44 (3.54%)	
No	762 (67.43%)	915 (71.76%)	821 (67.46%)	831 (66.80%)	
**Coronary heart disease**					0.01
No	91 (8.05%)	103 (8.08%)	108 (8.87%)	148 (11.90%)	
Yes	1039 (91.95%)	1172 (91.92%)	1109 (91.13%)	1096 (88.10%)	
**Prostate enlargement**					0.001
No	950 (84.07%)	1027 (80.55%)	949 (77.98%)	922 (74.12%)	
Yes	180 (15.93%)	248 (19.45%)	268 (22.02%)	322 (25.88%)	

Mean ± SD for continuous variables, *P*-value was by survey-weighted linear regression. % for categorical variables, *P*-value was by survey-weighted Chi-square test. Q1 represents the unhealthiest diet quality, Q4 represents the healthiest diet quality. *P* < 0.05 presents significant difference.

### The association between HEI-2015 and PE

Multivariate regression analyses are shown in Table 2. Compared with Q1 of HEI-2015, HEI-2015 was positively associated with PE (Odds ratio [OR] = 1.016; 95% Confidence interval [95%CI] 1.008 to 1.024, *P* < 0.001). In addition, all three quartiles of HEI-2015 were correlated to PE in Model 1. However, no significant differences were found in the other two adjusted models.

**Table 2:** Association of Healthy Eating Index 2015 (HEI-2015) with prostate enlargement, weighted

Exposure	Model1^a^ (OR, 95% CI), *P*	Model2^b^ (OR, 95% CI), *P*	Model3^c^ (OR, 95% CI), *P*
HEI-2015 (continuous)	1.016 (1.008,1.024) <0.001	1.004 (0.996,1.012) 0.257	1.000 (0.992,1.009) 0.931
Quartile (Q) of HEI-2015			
Q1	1.0	1.0	1.0
Q2	1.281 (0.976, 1.682) 0.08	1.149 (0.864, 1.527) 0.35	1.098 (0.827, 1.458) 0.52
Q3	1.496 (1.120, 1.998) 0.008	1.189 (0.885, 1.598) 0.26	1.093 (0.812, 1.471) 0.56
Q4	1.765 (1.339, 2.326) <0.001	1.170 (0.896, 1.530) 0.25	0.998 (0.764, 1.306) 0.99
*P* for trend	<0.001	0.28	0.97

a Non-adjusted model: adjusted for None.

b Minimally adjusted model: adjusted for age and race.

c Fully adjusted model: adjusted for age, race, education level, family income-to-poverty ratio, marital state, BMI, smoke, alcohol, energy, diabetes, hypertension, coronary heart disease. *P* < 0.05 presents significant difference.

Furthermore, the association between HEI-2015 components (continuous) with PE was investigated (Table 3). The adequacy components (total vegetables, total fruits, whole fruits, whole grains, total dairy) showed a positive correlation with PE in Model 1 (*P* < 0.001). The moderation component (Sodium) showed a negative correlation with PE (*P* = 0.02) in Model 1. In the primary adjusted model, total vegetables, total fruits, whole fruits, and total dairy have the same trends as Model 1. In the fully adjusted Model 3, higher vegetables intake (OR = 1.073; 95%CI 1.015 to 1.134, *P* = 0.02) and higher total dairy intake (OR = 1.034; 95%CI 1.009 to 1.061, *P* = 0.01) were significantly associated with higher risk of PE. Added sugar was negatively related to PE risk only in Model 3 (OR = 0.967; 95%CI 0.938 to 0.996, *P* = 0.03). There were no significant differences in other HEI-2015 components.

**Table 3:** Association of Healthy Eating Index 2015 (HEI-2015) components with prostate enlargement, weighted

Components	Model1^a^ (OR, 95% CI), *P*	Model2^b^ (OR, 95% CI), *P*	Model3^c^ (OR, 95% CI), *P*
**Adequacy components**			
Total vegetables	1.123 (1.065,1.184) <0.001	1.072 (1.016,1.130) 0.01	1.073 (1.015,1.134) 0.02
Total fruits	1.138 (1.096,1.181) <0.001	1.042 (1.001,1.085) 0.048	1.022 (0.979,1.067) 0.30
Whole fruits	1.134 (1.094,1.175) <0.001	1.056 (1.015,1.100) 0.01	1.037 (0.996,1.081) 0.08
Greens and beans	1.018 (0.975,1.063) 0.41	1.039 (0.992,1.087) 0.11	1.034 (0.985,1.085) 0.17
Whole grains	1.071 (1.039,1.105) <0.001	1.013 (0.981,1.046) 0.44	1.003 (0.966,1.042) 0.86
Total Dairy	1.059 (1.038,1.080) <0.001	1.038 (1.015,1.061) 0.002	1.034 (1.009,1.061) 0.01
Total protein foods	1.001 (0.915,1.096) 0.98	1.025 (0.930,1.130) 0.63	1.012 (0.916,1.118) 0.80
Seafood and plant proteins	1.006 (0.964,1.049) 0.80	1.010 (0.966,1.055) 0.68	0.996 (0.952,1.043) 0.87
Fatty acids	1.005 (0.977,1.034) 0.72	1.011 (0.982,1.041) 0.46	1.011 (0.980,1.042) 0.49
**Moderation components**			
Sodium	0.971 (0.947,0.996) 0.02	0.976 (0.951,1.001) 0.06	0.973 (0.944,1.002) 0.07
Refined grains	1.004 (0.981,1.028) 0.74	0.993 (0.968,1.019) 0.61	0.986 (0.959,1.014) 0.31
Saturated fats	0.983 (0.952,1.014) 0.29	0.985 (0.953,1.018) 0.37	0.985 (0.951,1.020) 0.39
Added sugars	1.016 (0.991,1.042) 0.21	0.980 (0.953,1.007) 0.14	0.967 (0.938,0.996) 0.03

a Non-adjusted model: adjusted for None.

b Minimally adjusted model: adjusted for age and race.

c Fully adjusted model: adjusted for age, race, education level, family income-to-poverty ratio, marital state, BMI, smoke, alcohol, energy, diabetes, hypertension, coronary heart disease. *P* < 0.05 presents significant difference.

### Subgroup analysis

The results of subgroup analyses indicated that race, education level, and smoke were effect modifiers for the association between HEI-2015 and PE in the fully adjusted model (*P* < 0.05) (Table 4). Based on, there were significant differences in Mexican American, non-Hispanic black, non-Hispanic white, other Hispanic, and other races (OR = 1.027, 1.003, 1.000, 0.964 and 1.004) with interactive *P* = 0.01. The odds of stratified educational levels of less than 12th grade, high school grade, and college graduate were 1.019, 1.004, 0.994 individually, with *P* of 0.049 for interaction. In addition, the odds of non-smoker and smoker were 0.989 and 1.007 individually, with *P* of 0.02 for interaction.

**Table 4:** Stratified logistic regression analysis to identify variables that modify the correlation between Healthy Eating Index 2015 (HEI-2015) and prostate enlargement, weighted

Stratified	OR (95% CI)^a^	*P*	*P* for interaction
**Age**			0.20
40 ≤ age ≤ 50	0.994 (0.973, 1.015)	0.57	
50 < age ≤ 60	1.003 (0.986, 1.019)	0.76	
60 < age ≤ 70	0.997 (0.984, 1.011)	0.67	
70 < age ≤ 80	1.000 (0.986, 1.015)	0.96	
80 < age	1.024 (1.001, 1.048)	0.05	
**Race**			0.01
Mexican American	1.027 (1.008, 1.047)	0.01	
Non-Hispanic Black	1.003 (0.988, 1.018)	0.71	
Non-Hispanic White	1.000 (0.991, 1.010)	0.92	
Other Hispanic	0.964 (0.934, 0.995)	0.03	
Other Race	1.004 (0.937, 1.075)	0.92	
**Education level**			0.049
Less Than 12th Grade	1.019 (1.001, 1.038)	0.04	
High School Grade	1.004 (0.991, 1.018)	0.53	
College Graduate	0.994 (0.983, 1.004)	0.27	
**BMI (kg/m** ^2^ **)**			0.05
BMI ≤ 20	0.930 (0.881, 0.981)	0.01	
20 < BMI ≤ 25	1.005 (0.990, 1.021)	0.51	
25 < BMI ≤ 30	1.000 (0.989, 1.011)	0.98	
30 < BMI	1.002 (0.988, 1.015)	0.81	
**Smoke**			0.02
Non-smoker	0.989 (0.976, 1.001)	0.09	
Smoker	1.007 (0.997, 1.016)	0.17	
**Diabetes**			0.11
DM	0.984 (0.969, 0.999)	0.05	
IFG	0.990 (0.960, 1.020)	0.50	
IGT	1.003 (0.973, 1.033)	0.86	
No	1.006 (0.996, 1.017)	0.26	
**Hypertension**			0.70
No	0.999 (0.987, 1.011)	0.82	
Yes	1.002 (0.992, 1.012)	0.76	
**Coronary heart disease**			0.27
No	1.002 (0.994, 1.010)	0.63	
Yes	0.989 (0.966, 1.012)	0.35	

a Adjust for adjusted for age, race, education level, family income-to-poverty ratio, marital state, BMI, smoke, alcohol, energy, diabetes, hypertension, coronary heart disease. *P* < 0.05 presents significant difference. All the models are not adjusted for the variable itself in each stratification.

## Discussion

In this cross-sectional study, we explored the association between HEI-2015 and the prevalence of PE from 4-year cycles (2001–2008) based on the NHANES database. In general, no obvious association of quartiles of HEI-2015 scores and PE prevalence in US adults was identified in adjusted models. Notably, of the 13 components of HEI-2015, total vegetables and fruits might play a positive role in the risk of PE. In addition, race, education level, and smoke are able to modify the relation.

Diet was thought to be important ranging from BPH to prostate cancer (15, 16). Studies have reported that intake of fruits, vitamins, and protein was recognized as a protective factor in BPH prevalence. However, excess intake of lipid and red meat contributed to BPH (6). For HEI-2015 score, higher consumption of adequacy components (i.e. total vegetables, total fruits, and dairy) and lower consumption of moderation components (i.e. sodium, refined grains, and added sugars) indicated a more balanced and healthier diet. Our studies demonstrated that enough intake of total vegetables and total dairy might be associated with a higher risk of PE.

To our knowledge, vegetables have long been identified to inverse PE. For example, a prospective study revealed a lower consumption of vegetables in men with BPH (11). In addition, fruits were not associated with the prevalence of BPH in some previous studies. However, a study showed an inverse relation between fruits intake and risk of BPH (17). However, our study reported an inverse relation between vegetable and fruit intake and the risk of PE. Although significant, the differences were minor compared with the crude model with a decreasing trend. Most evidence indicated that increased vegetable intake can reduce the prevalence of BPH. Interestingly, a recent multicenter in China demonstrated that the prostate volume enlarged as the age and vegetable intake increased (18). In fact, PE was highly associated with older age. In the baseline data, the number of participants aged 60 years old or lower decreased along with the increase of HEI-2015 score. The participants aged 60 years old or older seemed to have a healthier diet. Herein, the balance of age and vegetable intake might be the reason. Although older US population might have a healthier diet, the higher effect of aging on PE overwhelmed the effect of vegetable intake.

Milk and dairy products are main sources of many nutrients for humans including protein, calcium, magnesium, vitamins, pantothenic acid, and others (19). Dairy products are crucial components in preventing physical frailty (20). Nearly all the evidence supported the fact that dairy products intake helps meet nutrient recommendations to prevent some specific diseases (21). However, studies demonstrated that a higher dairy intake is associated with the risk of prostate cancer (22). A systematic review reported that the risk ratio of dairy intake is higher in prostate cancer with high heterogeneity of 77.1% (23). Therefore, the effect of dairy products on prostatic diseases is uncertain. To date, there was still no adequate evidence to ensure the relationship between dairy intake and the risk of PE. The frequent intake of meat and dairy products was identified as an increased risk factor for BPH (24). In our study, the dairy intake was positively correlated with the risk of PE which was consistent with the previous study (25). For the inconsistent results of dairy products intake, the minor, moderate, or excessive intake of dairy might have different effects on the risk of BPH. Moreover, dietary fat in milk might also be the problem (26). Further studies with different amount of dairy products consumption are needed for researching in depth.

Furthermore, we found that race, education level, and smoking could modify the association between HEI-2015 and PE. A higher education level and being a non-smoker resulted in a lower risk of PE. In contrast, the risk of PE is much higher in the Mexican American population. Therefore, we concluded that higher education and non-smoker population were more likely to have a higher HEI-2015 score. A higher education level indicated a higher diagnostic rate of BPH (27). However, the hormone level and the diet components of being Mexican American contributed to a higher risk of BPH. Interestingly, the adults aged between 40 and 60 years old had a lower HEI-2015 score in Q4 than those above 60 years old. We guessed that elder adults might focus more on healthy diets than younger ones, and had a higher HEI-2015 score. Therefore, the HEI-2015 showed the same trend with the prevalence of BPH. Higher rates of diagnosis lead to a relatively higher ‘risk’ of BPH.

Our study is a large-scale analysis based on the NHANSE data set to explore the association between HEI-2015 components and the risk of PE. HEI-2015 is the latest version of HEI in evaluating nutrition intake. Previous studies were restricted to androgen and aging. However, little solid evidence can elaborate the exact mechanisms of BPH. Since a novel trend in nutrition research triggered the attention, NHANES provided adequate diet intake data of the US population to evaluate the association between diet and diseases. Herein, our study can serve as a novel insight into the prevention on BPH. Furthermore, the HEI-2015 components, especially vegetables and dairy products intake, provided a contradictory suggestion to other studies that still need more research for validation.

There are some limitations of our study. First, given that NHANES is a cross-sectional study, we cannot depict the accurate association between HEI-2015 and the risk of PE. Second, we collected the data of the first 24 h recall to gain more intact data, which cannot represent all the daily nutrition intake well. The associations of vegetable and dairy intake with PE may be affected by type I error, which needs further validation. Third, all the diet and PE information were obtained from interview. Hence, there were inevitably some missing data or unpredictable responses in interview, causing a bias in analysis. Additionally, the enlargement of the prostate cannot fully stand for BPH. Furthermore, age was identified an important factor affecting the prevalence of BPH. Although we adjusted possible confounding factors to balance the regression analysis, there were still some unknown variables that influenced the final results.

## Conclusions

Our results from the NHANES database revealed no significant difference between HEI-2015 and the prevalence of PE after full adjustment. Total vegetables and dairy product are synergistically correlated with the risk of PE. Given the current findings and limitations, further studies are needed for validation.

## Data Availability

Publicly available datasets were analyzed in this study. This data can be found at: https://www.cdc.gov/nchs/nhanes/.
